# Attitudes towards and Beliefs about HIV Testing among Latino Immigrant MSM: A Comparison of Testers and Nontesters

**DOI:** 10.1155/2013/563537

**Published:** 2013-12-23

**Authors:** Rosa Solorio, Mark Forehand, Jane Simoni

**Affiliations:** ^1^Department of Health Services, School of Public Health, University of Washington, 4333 Brooklyn Avenue NE., P.O. Box 359455, Seattle, WA 98195, USA; ^2^Department of Global Health, School of Public Health, University of Washington, Seattle, WA 98104, USA; ^3^Social and Behavioral Prevention Core, Center for AIDS Research, University of Washington, Seattle, WA 98104-2499, USA; ^4^Foster School of Business, University of Washington, Seattle, WA 98105, USA; ^5^Department of Psychology, University of Washington, Seattle, WA 98195-1525, USA

## Abstract

Latino immigrant men who have sex with men (MSM) are at risk for HIV and delayed diagnosis. An exploratory study using qualitative interviews that assess the beliefs and attitudes of 54 Latino immigrant MSM in Seattle, Washington, is presented. The goal of this research is to determine whether attitudinal differences exist between participants who had and had not been tested and to use any insight into the development of a media campaign to promote testing. Over one-third of the men have never been tested for HIV. Nontesters are more likely to be men who have sex with men and women, have less knowledge about HIV risks, perceive their sexual behaviors as less risky, and deflect HIV-related stigma. Testers are more likely to be self-identified as being gays. Both groups believe that fear of a positive result is the main barrier to testing. Both groups believe that family members have negative attitudes towards HIV testing and that having Latino staff at HIV testing sites hinders confidentiality. Financial concerns with regard to the cost of testing were also expressed by both groups. Based on these insights, recommended strategies for the development of HIV prevention and testing campaigns are made.

## 1. Introduction

In the United States, Latino are disproportionately affected by HIV infection and have an HIV diagnosis rate that is three times that of non-Latino Whites [1]. An important public health issue among Latinos is that they tend to be diagnosed later in the course of HIV infection than nonLatino Whites [2] and present with lower CD4 counts and more opportunistic infections [3, 4]. In a recent systematic review of delayed HIV/AIDS diagnosis in the United States, Latino males and Latino immigrants were found to have the highest risk for late diagnosis [5]. Late HIV diagnosis has negative implications for individual morbidity and mortality and for public health. The HIV/AIDS epidemic can be lessened substantially by increasing the proportion of HIV positive persons who are aware of their status [6]; 70% of persons who find out that they are HIV positive stop having unsafe sex [7]. Importantly, timely diagnosis and treatment of HIV benefit individuals through a reduction in morbidity and mortality and offer public health benefits through a lowering of the viral load across a population, potentially slowing down new infections [8].

Low levels of acculturation to U.S. culture, defined by limited English proficiency and fewer than 5 years in the U.S [9–11], have been associated with late HIV testing. A previous study also indicates that Latinos who have a low income are less likely to report HIV testing [12]. A recent study in Washington State examined a decade of data on predictors of late HIV testing and found that Latino immigrants were persistently tested late compared to Latinos born in the U.S. [13]. These studies indicate that low income Latino immigrants with limited English proficiency may be in need of public health strategies to raise their awareness about the need for HIV testing.

In the USA, several HIV/AIDS mass communication campaigns have targeted English speakers, but only a few campaigns have targeted Latino MSM Spanish speakers [14]. In a review of the literature, we could locate only two radio campaigns in the USA. which have targeted Latinos with HIV testing messages; both are reported to have had limited impact on HIV testing [15, 16]. One study targeted Latinos at high risk for HIV, including transborder farmworkers, youth sex workers, and MSM and found that only 25% of people seeking HIV testing at sites promoted in campaign reported exposure to the campaign messages [15]. A second study targeted Latino men who have sex with men and women (MSMW) and offered health exams *inclusive* of HIV testing at local sites. This second study reported an 86.8% exposure rate for their sample of 98 MSMW and 85.9% exposure rate for their sample of over 900 heterosexuals. Despite these high levels of exposure, there was no impact on HIV testing rates among the MSMW when comparing baseline, campaign, and postcampaign HIV testing rates [16]. Recently the U.S. Centers for Disease Control and Prevention has developed an HIV campaign for Latinos (http://hivtest.cdc.gov/reasons/) that focuses on “reasons” for getting an HIV test; the evaluation of this campaign is pending.

To successfully reach Latino immigrant MSM with messages that promote HIV testing, it is important to first understand their beliefs and attitudes towards HIV testing. Although such a focus on Latino MSM has not yet been conducted, a recent systematic review of qualitative evidence on HIV testing among MSM in high income countries reports that (a) uncertainty about HIV status is an important motive for testing (although denial is a common response to such uncertainty); (b) fear of the consequences of a positive test is widespread and may take several forms; (c) a sense of responsibility towards oneself or one's partner may be a motive for testing; (d) the perceptions of stigma, from other gay men or from the wider culture, is a barrier to testing; and (e) gay and other MSM have clear preferences regarding testing services; specifically, gay and other MSM have a strong preference for confidential, nonjudgmental, and gay-positive service providers [17].

The purpose of this study is thus to conduct qualitative interviews with Latino immigrant MSM monolingual Spanish speakers and examine their beliefs and attitudes towards HIV testing, including their perceived barriers and facilitators associated with testing. We are especially interested in seeing if differences emerge among testers and nontesters so that a future campaign may target the nontesters. The data will be used to inform the development of a media campaign that will target Latino immigrant MSM Spanish speakers with messages that promote timely HIV testing.

## 2. Methods 

We conducted in-depth qualitative interviews with Latino MSM from May 2009 to March 2010 in Seattle, Washington. This methodology provides an opportunity to investigate participant reaction to HIV testing more fully than do methodologies that collect data from participants using quantitative surveys with predefined response options. In addition, the use of individual interviews allows the participants to have more privacy and facilitates their sharing of sensitive information compared to group settings. We used a grounded theory approach with open coding to generate lists of emergent salient factors according to participant's experiences and perspectives [18].

A recruiter approached potential subjects as they exited in Entre Hermanos, a community-based organization serving the health needs of the Latino lesbian, gay, bisexual and transgender (LGBT) community in Seattle, WA. In addition, participants were recruited through fliers distributed at community sites and health care organizations in King County, WA; interested parties were able to call in into a study telephone number and inquire about study. For this study, 39 participants were recruited at Entre Hermanos and 15 through flyers. Entre Hermanos permitted project staff to conduct the interviews with clients of their organization on site, in a private room. The project staff explained that the purpose of the interviews was to gain an understanding of barriers to HIV testing to inform a media campaign to promote HIV testing among Latino MSM.

Men were eligible for the study if they were biologically male, of Latino/Latino descent, between the ages of 18–40 years, Spanish speaking (i.e., monolingual), and had a history of having sex with men in the past 12 months. MSM who had ever had sex with a woman were also eligible to participate. Persons with obvious mental or cognitive impairments or under the influence of drugs or alcohol at the time of screening were excluded from study participation (only one participant was excluded for this reason). This study was approved by the University of Washington Institutional Review Board.

A total of 54 Latino MSM participated in the in-depth interviews. We developed a semistructured interview guide that included some of the domains used in the AIDS Risk Reduction Model [19], including individual, health system, and counseling and testing factors, to inquire about the participants' HIV testing behaviors. Participants were asked questions that included demographic information, self-described sexual orientation, family background, health seeking behaviors, HIV knowledge, sexual risk behaviors, self-perceived HIV risk, HIV testing history, and whether cultural factors influenced their decision to seek HIV testing; if the men had never been tested, they were asked if they had ever thought about testing, and if so, the reasons for deciding not to undergo testing and whether they had intentions to test in the next 12 months (see Appendix for Interview Questions). Participants were also asked to make recommendations on how to improve HIV testing rates among Latinos: they were asked “If you had a friend who needed HIV testing, what do you think might help him in seeking and accepting testing?” They were also asked for their recommendations on how to develop a culturally tailored media campaign for Latino MSM to promote HIV testing: they were asked “If we were to create a campaign to promote HIV testing among Latino MSM, how would you recommend that we develop it?”

Two trained interviewers conducted the interviews. Each participant was paid $25 for participating in a 1.5-hour interview. All interviews were digitally recorded, transcribed verbatim, and imported into ATLAS.ti [20]. All data were de-identified to protect confidentiality. The data was analyzed using the tenants of grounded theory [21]. In practice, this entails a thematic analysis of the transcripts from which “categories” and “subcategories” were created. A process of constant comparison between interviews was used to consolidate dominant themes and data collection ceased when “saturation” was achieved, with themes and scenarios being repeated in successive interviews. All interviews were independently coded in Spanish by two researchers. Coding differences were identified and discussed until consensus was reached. Coding was done in Spanish and later translated to English. All survey instruments were pretested with Spanish speaking MSM to assure that questions were clear and easy to understand.

We used simple coding and retrieval to categorize emergent themes and concepts. Barriers were defined broadly as any factor that interferes with HIV testing. The analysis was conducted using ATLAS.ti [20].

## 3. Results 

Most of the participants were of Mexican descent (80%), had an age range from 18 to 40 years with the median age of being 25 years, and had annual incomes below $20,000. All participants were monolingual Spanish speakers and most had resided in the USA. for less than 5 years and had less than a high school education; thus, the sample was a low-income one with low levels of acculturation to U.S. culture and low levels of education. Most of the men (60%) were clear about their self-described sexual orientation with 28 identifying themselves as gay, 2 as transgender, and 2 as bisexual; however, the other 40% explicitly stated that they were not gay and amongst these, 13 described a history of having sex with men and women and 9 described a history of having sex with men only. Some of these men asserted their male masculinity based on practicing only insertive anal sex with men. Among the 54 men, 35 had been tested for HIV (65%) and among these, 7 report positive results (20% of testers); 35% of the sample has never received HIV testing. Most men were aware about the existence of blood tests, but only a few were aware about rapid HIV tests that use saliva and that such testing may provide results within 20 minutes. About one-third of the sample could not identify a place for HIV testing in King County, WA.

When examining testers only, four main reasons emerged for undergoing testing: (a) men developed symptoms suggestive of HIV (i.e., pneumonia) and then saw a physician who recommended testing; (b) men sought testing after hearing rumors that a previous partner had tested HIV positive; (c) men were encouraged by a sexual partner to undergo testing so that unprotected sex could take place; and (d) men had a friend who was knowledgeable about HIV testing and who recommended that they undergo testing; sometimes this friend was someone from a local community-based organization who offered education on HIV testing. Among those who had received testing, only a handful received regular testing (i.e., every 6–12 months). Most men who had been tested reported feeling under severe stress when awaiting test results due to the possibility of testing positive, and a handful of men reported not returning for test results due to fear about a positive result and therefore not knowing their HIV status. Only two men expressed a perceived benefit from knowing one's status to reduce the chances of transmitting HIV infection to others. Another handful of men reported to undergoing testing, based on their sexual partner's suggestion, so that they could have unprotected sex.

When examining nontesters only, the main reasons for not seeking testing are as follows: (a) the men feel healthy and do not think that HIV testing is necessary; (b) the men perceive themselves to be at low risk for HIV because they believe that the type of sexual behaviors that they engage in do not place them at high risk for HIV (i.e., some men tended to have sex primarily with women and only occasionally with men, some men only engage in insertive anal sex with men, and some men only engage in oral sex with men); (c) they perceive their sexual partners to be healthy based on appearance. The low levels of perceived risk appear to be due to a combination of factors, including lower levels of knowledge about HIV risk and denial of HIV risk due to HIV-related stigma deflection; several MSMW asserted their male masculinity and asserted their self-described sexual orientation as not being gay and made statements that “only gay men get HIV.” When asked about intentions to undergo HIV testing in the next 12 months, much ambivalence was expressed. The participants expressed the following about HIV knowledge and self-perceived HIV risk:

*I feel healthy. On some occasions I have not used protection.*

*I do not have much knowledge about HIV. I do not use condoms. I just found out that HIV may be transmitted through oral sex.*

*Sometime I engage in sex work, sometimes with older white men who pay well. I know that you get HIV from having sex with infected people, but do not really know much else. I do not use any type of protection.*

*I do not believe that I'm at risk for HIV. I am not gay. Only the gay men get HIV. I have never had receptive sex, only insertive. I only used a condom once and did not like it. *

*I do not think that I will become infected. I believe only those who engage in receptive [anal] sex will get it. None of my partners have looked ill.*

*I am 100% active (Spanish term for active referring to engaging only in insertive anal sex).*

In comparing testers versus nontesters, differences emerged. Nontesters are more likely to be MSMW (40%) than testers (14%), to have less knowledge about HIV risks, to perceive their sexual behaviors as less risky, and to deflect HIV-related stigma with statements such as “I'm not gay” and “only gay men get HIV.” Nontesters are less likely to self-describe their sexual orientation as being gay compared to testers (20% versus 69%). The nontesters described their sexual orientation in the following ways:

*I am not gay. I have a wife and children.*

*My current partner is a woman. I do not behave like a gay man.*

*I have sex mostly with women and have some adventures with men.*

*I only have sex with men.*

As for reducing risk, the majority of the participants in both groups state that they would prefer to have a long-term relationship with their male partner but that such a partnership is hard to find because the men they meet do not seem to want this. Over 80% of the sample reports to have occasional partners. In addition, a handful of men report to live with a partner who is HIV positive; all of these men report always using condoms. In addition, about 10% of the sample reports engaging in sex with white men; sometimes, these are older white men who pay for sex. Among the handful of men who report engaging in sex work, some report always using condoms but others do not.

Both groups believe that *fear of a positive result* is the main barrier to testing and this belief supersedes all others. In such a situation, the men fear that their sexual partners, friends, and family would find out about their HIV status and that they would then face HIV-related stigma and rejection, including being called names, like “Sidoso” (Spanish term for a person who is “full of AIDS”), “Joto” (Spanish term for queer or faggot), and “Maricon” (Spanish term for effeminate man). Some men report suffering from anxiety just from thinking about HIV testing. Due to fear and apprehension about HIV testing, many men delay testing indefinitely.

*It is fear of being singled out as a “Sidoso.”*

*I think that it is fear. If you are HIV positive, society will single you out and therefore, I'm better off not testing.*

*If I tested positive, I would not tell my [sexual] partner…*

*I fear dying from HIV. I would be judged by the whole world for being promiscuous. For a very long time, I've lived traumatized, listening to the television and the radio…I used to suffer from panic attacks thinking that I had to get an HIV test…I felt crazy thinking about HIV due to a lack of sufficient information.*

Both groups cite the cultural factor of *familism* as a prominent barrier to HIV testing. Both groups expressed negative attitudes from family members towards HIV testing and feel that the simple act of seeking testing would be interpreted by family member as a confirmation of being gay or of being promiscuous. Although most men report that they think that their family members know that they are gay, most said that this was an issue that was not openly discussed. Men stated that family members, especially, would question why they would need an HIV test and they did not want to have to explain. Feared homophobia, on the part of family members, was expressed.The participants illustrate these fears with following statements:

*I'm afraid of being rejected by my family because if you have HIV, you are gay and that is it. People do not undergo HIV testing due to fear of being called a “Maricon.”*

*If family finds out that that one has undergone HIV testing, to them this means that one is a “Joto.”*

*If my family finds out about HIV testing, they will think that I'm promiscuous or that I have AIDS or worse.*

In addition, both groups believe that having Latino staff at HIV testing sites hinders *confidentiality. *This is an especially significant problem because the men fear that their families and/or friends will find out that they were seeking HIV testing and be perceived as “high risk” for HIV and they especially fear testing positive and other people in their community finding out about their status. The men expressed the following concerns:

*In places where HIV testing is offered, there are too many Latino staff and people say that they tell other people about one's HIV status. I think that is why Latinos do not go to those places.*

*It is difficult to get an HIV test. People want privacy and the testing places are not private. *

*People are embarrassed that someone they know will see them at an HIV testing site.*

Both groups also expressed financial concerns with regard to HIV testing. Several men stated that HIV testing is expensive and wanted information about where to go for free HIV testing. Financial concerns about the cost of treatment, if testing positive, were also commonly expressed:

*It might be easy to get an HIV test but it is expensive.*

*My main fear is that I will turn positive and that I will not be able to pay for the costly treatment.*

Other barriers to testing in both groups included other *cultural factors*, such as fatalism, religiosity, and machismo. Some men expressed fatalistic beliefs about what a positive HIV test result would mean (i.e., an imminent and painful death), as well as expressing their beliefs about religion's condemnation of homosexual behavior and machismo's role in keeping some men from seeking HIV testing. The men said the following about the cultural factors of fatalism, religion, and machismo:

*Fatalism*

*It is the worst fear because people say that a death from AIDS includes wasting and pain.*

*I fear death, becoming ill, like those I see on television…I do not have a doctor.*

*Yes, everybody is afraid to die from HIV but more so among those who are gay.*

*I have been to the hospital for testing…I did not return for the results…due to fear of results being positive…if I were to test positive, I may become mentally ill from thinking that I'm going to die.*

*I imagine that sooner or later it [HIV] would be inevitable.*

*Religiosity*

*According to religion, it is bad to be gay. HIV is God's punishment.*

*Religious beliefs keep people from seeking testing. I have fear and think that because I'm gay, I'm going to have HIV. I've had testing and know that I need to continue doing it but I am afraid.*

*Machismo*

*When it comes to HIV testing, it does not matter how one identifies, gay, bisexual or heterosexual. All that matters is whether one is into machismo…*

*I think it is both, machismo and fear that keeps people from testing.*

*I know about some gay friends who sleep around with those who are not gay and it is those guys who do not want to undergo testing; these are the machos. *

### 3.1. Recommendations for How to Get a Friend Who Refuses HIV Testing to Accept Testing

Participants who previously had positive experiences with HIV testing tend to recommend HIV testing to their friends. The participants recommend using the following strategies to get a friend to undergo testing:

*I would help him by accompanying him to a place where he could get more information (about HIV testing).*

*I would advise him to undergo testing because if he has it, although it is not curable, there are medications and this allows one to live better and longer.*

*HIV testing is beneficial because if one tests negative, one can take precautions, and if one tests positive, then there is treatment.*

*I would explain that an HIV test is important. It is best to not delay. It is not like a cold that is going to pass. It is a virus that stays inside of you…the truth is that there is no cure but there is treatment. I would offer my friend solutions to perceived problems. Most of the time, people defer testing due to fear.*

The participants who had not undergone HIV testing had the following recommendations to get a friend to undergo HIV testing:

* This is a very delicate subject (HIV) and one would need to approach it with great empathy.*

*I would try and find out about places where one could go without anyone knowing about it.*

*I would try and get more information for him and look for a testing site without Latino staff.*

*I would try and convince him without using technical words, like CD4 counts, because sometimes this discourages people and instead they get scared.*

*I would tell him about a testing site that offers free HIV testing.*

*I would try and find him a good psychologist to reduce fear about HIV first.*

### 3.2. Recommendations for How to Develop a Media Campaign to Promote HIV Testing

The testers were more enthusiastic about making recommendations to improve HIV testing among Latinos than the nontesters. However, the recommendations made were similar from both groups. The men want a campaign to use Spanish language, and they want to hear HIV prevention messages in radio, television, and the internet. They want the campaigns to focus on removing fear from HIV testing and to focus on the benefits. They want to hear HIV prevention messages that go beyond HIV testing and are inclusive of information about HIV testing sites, especially those that offer free testing and they want linkages to health care resources. They recommend using an empathetic peer model as a promoter of HIV testing. The following quotes are representative of their recommendations:

*Use Spanish Language and Emphasize Benefits from HIV Testing*

*I want to be spoken to in my own language, without formalities and to hear from someone who is truly knowledgeable.*

*The talk needs to be direct and focus on the benefits from testing.*

*Use Radio, Television, and Internet to Promote HIV Testing*

*Develop a radio program in Spanish…most Latinos listen to the radio. *

*Use the internet.*

*Use radio and television.*

*Raise Awareness about Free HIV Testing and Treatment*

*There needs to be more information, more publicity about places where one can go for free HIV testing and for treatment.*

*The campaign can raise awareness about HIV testing sites…about existing social support programs in the community [for Latinos]…given a phone number to call if they have questions…directing them to the internet [for more information]…if the HIV testing place mentioned is Gay City, then some will say, that place is only for gays…people need to know about other testing sites.*

*People need to know that if they undergo testing that free treatment exists.*

*Use an Empathetic Peer Model to Promote HIV Testing*

*The peer model needs to be empathetic.*

*The peer model needs to invite others to undergo testing…it must be done with love and knowledge.*

*It does not matter who the peer model is…what is needed is someone with knowledge about HIV.*

*A peer model with knowledge [about HIV] who could relate their personal story to convince others.*

*A peer model needs to be someone important who has credibility with Latinos.*

*Remove Fear*

*People need to be invited for testing without instilling them with fear and without the use of strange words…I've heard programs on the radio that promote HIV testing and they say to undergo testing before it is too late but instead, they should say, undergo testing and you might live a better life.*

*Due to our culture, if there is too much talk about HIV, instead of this motivating one to undergo testing, fear may develop and keep a person from testing. Fear needs to be removed…HIV talk needs to be smooth.*

*The fear needs to be removed…the language should not use medical terms…I have attended HIV talks and have found it difficult to understand the language used.*

*Latinos need to know that places exist for testing where they will not face discrimination and will not be judged. The fear that Latinos have is to be rejected for being HIV positive…fear of being judged.*

*HIV Prevention*

*It [campaign] should not be just about education but about raising people's consciousness that if they continue to have sex without condoms, then the sickness [HIV] will continue to be transmitted and it will never stop. *

*People need to know how to prevent HIV.*

*It [campaign] should emphasize the importance of maintaining one's health.*

*Promote testing for everyone, not just gay men…*

*I wish that people would understand more about HIV risks so that they would be more willing to use condoms.*

## 4. Discussion/Conclusion

This qualitative research identified important beliefs and attitudes towards HIV testing among Latino immigrant MSM. Important differences in attitudes and beliefs are found in comparing testers versus nontesters. Nontesters are more likely to be MSMW, to have less knowledge about HIV risks, to perceive their sexual behaviors as less risky, and to deflect HIV-related stigma. The main barrier for HIV testing for both groups, however, was the belief that if they undergo HIV testing and the result is positive, then they would face HIV-related stigma and rejection from friends and family members. The men reported that family members equate HIV testing with being gay and being promiscuous. Other significant barriers include confidentiality concerns and financial concerns about the cost of HIV testing. These findings may guide the future development of a mass media campaign to promote HIV testing among MSM and highlight the need for such a campaign to target MSM who are not identified as gays including MSMW and MSM who are not identified as gays.

It may be that MSMW and MSM who do not identify themselves as gays perceive themselves to be at low risk for HIV because they have less knowledge about HIV risk behaviors and/or perhaps they may perceive themselves to be at low risk because they are in denial about being at risk for HIV. The denial of risk may be due to stigma deflection; a stigma control technique has been previously described in which an individual passes as nonstigmatized by avoiding any and all things which may reveal their stigmatizing condition [22], in this case HIV risk behaviors. Since its discovery, HIV has been intimately associated with its primary forms of transmission: male homosexual contact and injection drug use. The stigma of HIV concerns these “socially deviant” transmission modes [23]. As a result, the socially disdainful risk behaviors become associated with individuals who are HIV positive or who simply acknowledge their own risk, hence the stigma of HIV/AIDS [24]. Previous qualitative studies that have examined barriers to HIV testing among MSM have not differentiated between MSM and MSMW [17]. Future research, using quantitative methods, may be helpful in assessing if it is the lack of knowledge and/or stigma that keep Latino MSM who do not identify themselves as gays from seeking timely HIV testing.

The finding that the nontesters tend to be MSMWs has long been suspected and this study confirms that suspicion. Future campaigns need to target this population with HIV prevention messages. One way of doing this is for campaigns to promote HIV testing towards MSM who are not identified as gays (this group may include MSMWs or other MSM who are not identified as gays). Future campaigns need to offer such MSM accurate information about risk behaviors in a sensitive manner; such campaigns will need to be created for MSM who are not identified as gays and therefore imagery and content specific to this population will be necessary.

Both testers and nontesters stated that the main barrier to HIV testing is fear. Many types of fear were expressed, including fear of testing positive and then having to face HIV-related stigma from family members, partners, and friends; fear about homophobia on the part of family members; fear about not being able to afford treatment; and fear about an imminent and painful death. The fear of testing positive is consistent with previous research on other populations of MSM (including those who are White [25]), and so is the HIV-related stigma [26]. Many of the men fear the homophobia that their family members express; homophobia among Latino families has been previously described [27]. The fear of being rejected by family members simply for seeking HIV testing or for testing HIV positive is a significant barrier for undergoing testing. Previous studies on Latino MSM have not reported on the derogatory terms that men fear being called, like “Sidoso”, “Joto,” and “Maricon”. Such fears will need to be addressed in future media campaigns to be able to promote HIV testing. The men who are identified as being gays believe that their family members know that they are gays but they state that this issue is not openly discussed; this finding is consistent with previous reports about the silence that exists in Latinos families with regard to human sexuality and homosexuality [28].

Both groups of men are very concerned about the lack of confidentiality that exists at HIV testing sites. This concern may be specific to racial/ethnic minorities in the United States. Some of the men believe that Latino staff at HIV testing places will disclose their results to other people in their Latino community. This is especially concerning when one considers that Latino Spanish speakers are an ethnic minority and they tend to live in segregated communities in the USA; thus, if an HIV testing site has Latino staff, there may be a high probability that the staff member lives in the same community as the Latino man being tested. Due to this possibility, some of the men expressed a desire to receive HIV testing from a person who is non-Latino. This finding is surprising and is an important one for public health agencies that offer HIV testing to consider when attempting to promote HIV testing among Latinos.

The men in this study were low-income and they expressed financial concerns about HIV testing costs. These cost barriers are similar to previous studies that have used quantitative methods and report a direct association between income and HIV testing among Latino immigrant men [12, 29]. While most large urban areas in the USA offer free HIV testing through public health clinic sites and community-based organizations, such sites may be problematic for Spanish-speaking populations if there are no staff who speak Spanish. While the participants in this study state a preference for non-Latino staff at HIV testing sites, they still need to be able to communicate their needs.

The pervasive fatalism expressed by the men in case of an HIV positive result (i.e., fear of an imminent and painful death) and the fear about not being able to afford treatment appear to be related. Many Latino immigrants tend to be low-income and lack health insurance [30, 31]. Treatment affordability is an important issue to be addressed by Health Care Reform for immigrants who reside in the USA legally.

Both testers and nontesters provided insightful recommendations for how to promote HIV testing. The men unanimously want campaigns to be in Spanish language. The men want the campaign to focus on reducing the fear that is associated with HIV testing; we found this recommendation to be very insightful. The men also recommend having an empathetic peer model to promote HIV testing. The men want media campaigns to raise awareness about free HIV testing sites and offer linkages to health care services for treatment.

One approach to promote HIV testing among Latino immigrant MSM may be to focus on lowering the barriers to HIV testing. This may be done by developing HIV prevention messages that counter the negative beliefs and reframe them with positive ones. For example, to address the fear associated with the possibility of testing HIV positive and the perception that it may be best to defer testing, a campaign may counter that the most likely outcome will be good news (i.e., more than 90% of Latino MSM who undergo HIV testing will have results that are negative [32] and therefore it is best to test now and practice safe sex to prevent HIV). It would also be important to counter the perception that a positive result means imminent death. This belief may be reframed by positioning HIV as a chronic disease for which treatment exists, allowing a person to potentially live a normal lifespan. In addition, to counter the lack of perceived benefit from testing, a focus on the benefits from timely HIV testing needs to be promoted (i.e., since treatment exists, individuals are better off knowing their status); such messages need to be conveyed in a way that would reach men who do not identify themselves as gays, especially MSMW.

To address the confidentiality concerns among Latino immigrant MSM, future campaigns may need to promote HIV testing sites at multiple locations, including medical and nonmedical sites and those within and outside of Latino communities. This way, the men may choose the site that meets their confidentiality needs. Some men will choose to be tested at a site that is far from their community and may even prefer a site that does not have Latino staff.

To address the financial concerns, a need exists for future campaigns to raise awareness about public health services that offer free HIV testing and about access to treatment under the Ryan White Act; in some states in the U.S. immigrants who are undocumented are eligible for treatment. In addition, to address the lack of perceived support that Latino immigrant MSM perceive from their families when seeking HIV testing, future campaigns may need to promote social support within existing Community-Based Organizations (CBOs). Also, since peer social support was found to be a facilitator for HIV testing in this study, future campaigns may promote HIV testing by encouraging the men to seek out peer social support from trusted friends and invite them to accompany them to HIV testing site. This may help reduce stress while they await results and may allow for continuation of social support, after testing.

These qualitative interviews generated rich data and have led to ideas for potential approaches for a media campaign as described above. Future research is needed to test these potential approaches to promote HIV testing with Latino MSM. However, as with most research studies, there are limitations to the current study that need to be discussed. First, most of the participants in this study are monolingual Spanish speakers of Mexican descent with less than five years living in the USA.; thus, the sample would be considered one with low levels of acculturation to U.S. culture and the findings would therefore be reflective of this population of immigrant Latinos. Second, a little over one-half of the sample was identified as being gays and almost one-fourth of the men were self-described MSMW who did not identify themselves as gays; since the sample was recruited from Entre Hermanos, a Community-Based Organization serving the LGBT community in Seattle, WA, it is possible that the MSMW sample is an underrepresentation of MSMW who may not be comfortable seeking services at this site. The study findings need to be interpreted keeping all of these factors in mind.

The rich data generated from this study will be used to inform the development of a mass media campaign to promote HIV testing among MSM who do not identify themselves as gays by reducing barriers to testing (i.e., reduce fear, promote safe sex, promote benefits from testing, promote multiple sites for HIV testing to address confidentiality concerns and promote free HIV testing to address financial concerns, and offer linkages to health care services and social support). Previous campaigns have mainly targeted Latino MSM who were identified as gays and have used different approaches to promote testing. Most campaigns to date have focused on alerting men of their high risk for HIV. Based on the current study findings, such fear tactics may be counterproductive. The “Tu No Me Conoces” campaign focused on raising awareness that one may not be aware about sexual partner's risk behaviors and therefore may be at high risk for HIV [15]. The “Hombre Sanos” campaign (translated to English means “Healthy Men”) promoted HIV testing and a physical exam, combined, to encourage the target population to go to a health center and receive both and thus promoted wellness [16]; both of these campaigns had limited impact on HIV testing. The CDC “Razones” Campaign (http://hivtest.cdc.gov/reasons/) is attempting to promote HIV testing by offering “reasons” for testing (i.e., a concerned aunt may care about the man's health and may want them to be healthy and to be tested); evaluation of this campaign is pending.

The next step for this current research is to take the qualitative findings and translate them into HIV prevention messages to be used in a media campaign to promote HIV testing among Latino MSM who are not identified as gays. The media campaign will involve consultation with a communication firm and marketing experts to enhance message development. The campaign will focus on promoting sexual health, including HIV testing and safe sex, and will be positioned as an outreach intervention that promotes HIV testing in an accepting environment and provides linkages to HIV testing sites, health care services, and social support.

## Appendix

### A. Interview Guide

#### A.1. Family Background

1. Where did you grow up? Tell me about your childhood and your family.
2. How close do you feel to your family?
3. Can you count on your family to help you in time of need? If yes, how does your family help you? If not, why not?
4. Are you in conflict with your family? If yes, does the conflict relate to your sexual orientation?
5. Could you describe in what ways you feel part of the Latino culture? American culture?

#### A.2. Health Seeking Behaviors

1. Describe to me any serious health problems that you had when you were younger?
2. How did you take care of them?
3. How do you take care of your health problems now?

#### A.3. HIV Knowledge

1. Tell me what you know about HIV infection? How is it transmitted? What are the health consequences?
2. Do you know anyone who is living with HIV? Tell me their story.
3. Do you understand the benefits from timely HIV diagnosis? How about the benefits from HIV treatment? Please explain these to me.
4. If a person wants an HIV test in the city of Seattle, do you think that is easy or difficult to find a testing site? Explain your answer.

#### A.4. HIV Risk and Culture

1. We know that people who put their health at risk by having unprotected sex are nervous about getting tested for conditions, such as HIV. We wonder about how “cultural topic inserted (i.e., familism, religiosity, and machismo)” might make it ever harder for Latinos.
2. What do you think about that “cultural topic?”
3. Does that make sense to you? Why or why not?
4. How might that work?…what is about “cultural topic” that might make it easier or harder for Latinos to get HIV testing?
5. If “cultural topic” does make it harder, what might be done to make it easier or more comfortable?

#### A.5. HIV Testing Experience

1. I'd like you to think back to the last time you got an HIV test. Take a minute to remember everything you can about it and then tell me the whole story, starting from what led up to the test, why you went to get it, and where it was done and then how you felt afterwards?
2. Tell me about the type of test. Who administered the test? How was the counseling? What was discussed in counseling? How did you feel after you left? What did you like or dislike about the experience. Were you concerned about privacy at all? How was the waiting period? When did you go back for the results? Who gave you the results?
3. How were the results explained/presented to you? What were your results? Were these the results you were expecting? Why did you expect these results? How did you feel after the results? What was discussed after you had the results?
4. Who did you share your results with? How did they react? Who did you not tell but would like to tell? Why did you not tell them?
5. When you first thought about getting an HIV test for the very first time, who was the first person that you discussed this with? Why this person? Do you consult with this person for other health problems?
6. How was this last test compared to other tests you've had, did you like it or not? Was it typical of other tests?
7. After this HIV test, did anything change in your life (drug use, sex, views on risk)?

#### A.6. Repeat Testing

1. How many times have you tested in your life? How often do you test? Do you test regularly? If so, why?

#### A.7. For Participants Who Have Not Been Tested for HIV

1. Have you ever thought about testing/or been approached about testing? What happened? How did you hear about it?
2. Why did you decide not to test?
3. What do you think would happen if you did test?
4. How likely is it that you will test for HIV over the next 12 months?

#### A.8. Participants Recommendations for Improving HIV Testing among Latino Men

1. If you had a friend who needed HIV testing, what do think might help him in seeking and accepting testing?
2. If we were to create a campaign to promote HIV testing among Latino MSM, how would you recommend that we develop it?
